# Nano-engineering the Antimicrobial Spectrum of Lantibiotics: Activity of Nisin against Gram Negative Bacteria

**DOI:** 10.1038/s41598-017-04670-0

**Published:** 2017-06-28

**Authors:** Marija Vukomanović, Vojka Žunič, Špela Kunej, Boštjan Jančar, Samo Jeverica, Rok Podlipec, Danilo Suvorov

**Affiliations:** 1 Advanced Materials Department, Jožef Stefan Institute, Jamova 39, 1000 Ljubljana, Slovenia; 2 Institute for Microbiology and Immunology, Medical Faculty, University of Ljubljana, Zaloška 4, 1000 Ljubljana, Slovenia; 3 Laboratory of Biophysics, Condensed Matter Physics Department, Jožef Stefan Institute, Jamova 39, 1000 Ljubljana, Slovenia

## Abstract

Lantibiotics, bacteria-sourced antimicrobial peptides, are very good candidates for effective and safe food additives. Among them, nisin is already approved by the EU and FDA, and has been used in food preservation for the past 40 years. Now, there is a possibility and strong interest to extend its applicability to biomedicine for designing innovative alternatives to antibiotics. The main obstacle is, however, its naturally narrow spectrum of antimicrobial activity, focused on Gram positive bacteria. Here we demonstrate broadening nisin’s spectrum to Gram negative bacteria using a nano-engineering approach. After binding nisin molecules to the surface of gold nano-features, uniformly deposited on spherical carbon templates, we created a nanocomposite with a high density of positively charged groups. Before assembly, none of the components of the nanocomposite showed any activity against bacterial growth, which was changed after assembly in the form of the nanocomposite. For the first time we showed that this type of structure enables interactions capable of disintegrating the wall of Gram negative bacteria. As confirmed by the nisin model, the developed approach opens up new horizons for the use of lantibiotics in designing post-antibiotic drugs.

## Introduction

Lantibiotics, proteinaceus toxins ribosomally synthesized by certain types of bacteria^1^, possess antibacterial activity against other similar or closely related bacterial strains. As a powerful and safe food additive, nisin is a member of these peptides, is on the positive lists of the EU (E234 food additive) and the FDA for food preservatives and has been approved and applied in food preservation in over 50 countries for a period of almost 40 years^2^. Because of the superior stability and more effective mechanism, these bacterial antimicrobial peptides are frequently regarded as very promising candidates for designing a new generation of naturally sourced antimicrobials^3, 4^. The mechanism they use is strongly dependent on interactions with bacterial membranes^5, 6^. Lantibiotics use different ways to kill bacterial cells: they bind to the lipid II, influence the bacterial cell-wall synthesis and inhibit bacterial growth and/or induce the formation of pores in the cellular membrane, which leads to the leaking of the cellular content and directly causes cellular death^7–9^. Nisin specifically forms a complex with the lipid-II wall precursor^10^ to influence the process of cell-wall synthesis and use it as a docking molecule for the formation of the 2-2-5-nm sized pores that contain the lipid-II molecule^11^ and are stable for seconds^8, 10^. The complexity of the mechanism and the dual mode of action used by antimicrobial peptides to exert their activity are responsible for their action against antibiotic-resistant species^7, 9^.

The main obstacle to the application of lantibiotics in clinical practice (including nisin as their representative) is their short half-life in the blood. In addition, they are limited by their narrow spectrum of antibacterial activity. Nevertheless, nisin has a potent antimicrobial activity against a wide spectrum of Gram positive bacteria, including *Staphylococcus aureus*, *Clostridium botulinum*, *Listeria monocytogenes*, *Bacillus cereus* and others, while Gram negative bacteria are protected by an outer, lipopolysaccharide membrane that shields the cytoplasmic membrane^2^.

Intensive work has been conducted to tailor the spectrum of antibacterial activity of lantibiotics^12, 13^. Standard strategies include destabilization of the outer membrane, by the bacteria pre-treatment using a short, sub-lethal heat shock, osmotic shock or by chelators (EDTA, citrates, lactoferrin etc.). After destabilization, the outer membrane of Gram negative bacteria is disintegrated and the bacteria become susceptible^14^. Another approach is through purification of the substance by removing salts, organic milk-sourced residuals and regulation of the pH^15^, since it was observed that salts decrease the permeability of the outer membrane, which blocks the activity against Gram negative bacteria^16^.

Recently, it was emphasized that the extraordinary properties of antimicrobial peptides, including lantibiotics, can be very effectively explored by the development of new technologies for their modifications, which will open up new ways for the re-initiation of their commercialization^17, 18^. New strategies comprise novel formulations of lantibiotics, including their loading into various drug-delivery systems or deposition/binding to the biologically applied surfaces^19^, which enhance their stability and provide advanced biological functions (an example is loading nisin together with Ag nanoparticles within PDLLA/PEO polymeric fibers)^20^. A very interesting approach also considers the molecular cloning of different tails (using antimicrobial peptides effective against Gram negative bacteria) fused to the *C* terminus of nisin, which enabled stronger activity in *E. coli* ^21^. Covalent grafting is particularly effective since it enables the immobilization of several antimicrobial peptides to the surface of a substrate, combining antimicrobial peptides with activity against Gram positive and Gram negative bacteria^22^. This approach also provides an enhancement of the antimicrobial activity of some nanomaterials (like multi-walled carbon nanotubes) and an amplification of their antimicrobial activity after the covalent immobilization of peptides, like nisin, to their surface^23^.

The impressive efficacy in antimicrobial action and the unique mechanism of antimicrobial activity typical for lantibiotics were the major motivations for us to design an innovative nano-engineering approach that will enable broadening the spectrum of antimicrobial activity of these peptides and exploit their perspectives for designing new, post-antibiotic drugs. Our work was driven by the hypothesis that a combination of the nano-architecture and the intensive positive charge provided by the high local density of nisin molecules will join in the destabilization of the outer membrane and the formation of the pores inside the rest of the bacterial membrane, which will enable the activity of nisin also against Gram negative bacteria. In this way we planned to develop a new technology capable of inducing the destabilization of the bacterial wall and provide activity across a broad spectrum of bacterial strains without the need for pre-treatments or purification before use.

## Results

**Figure 1:**
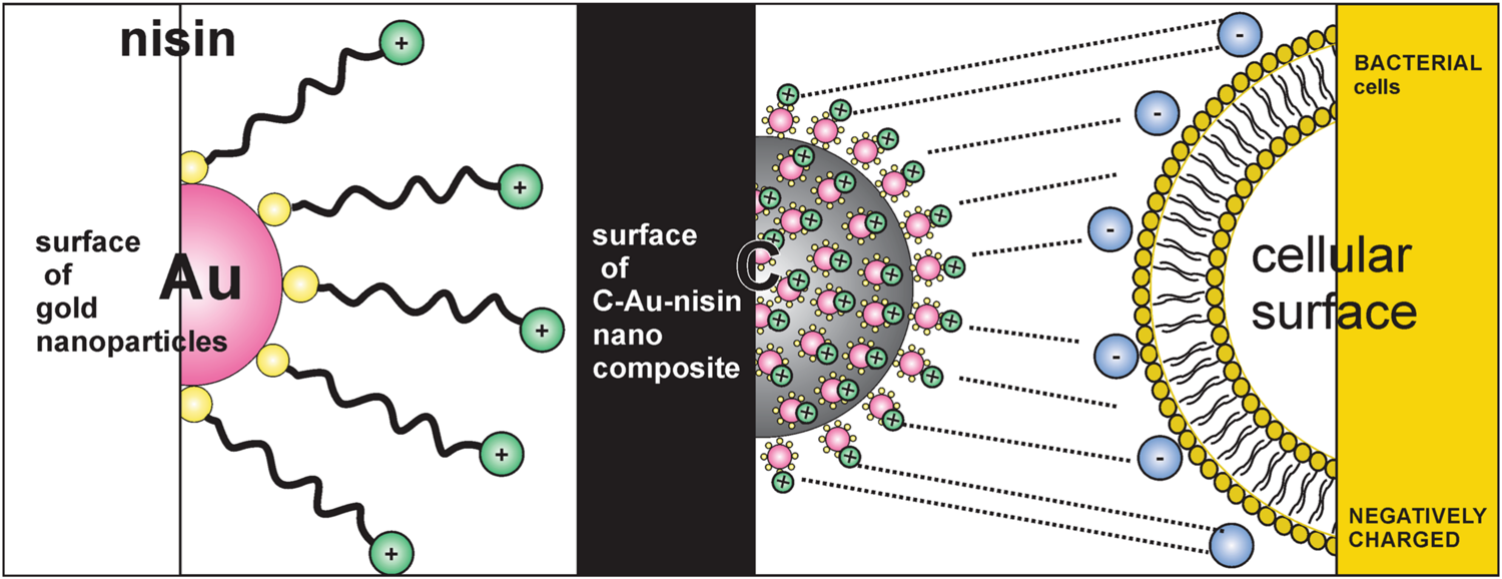
Illustration of the developed nano-engineering approach. Functionalization of the nisin at gold nano-features and uniform arrangement of gold nano-features on carbon-submicrospherical templates with an intensive positive charge available for an interaction with bacterial membranes.

The basic idea of the developed nano-engineering approach was to bind the nisin molecules to the surface of the arranged Au nano-features templated by carbon-submicrospheres (as illustrated in Fig. 1). The main goal was to obtain a high density of positively charged nisin peptide locally available for interactions with bacterial membranes.

### Nano-gold at Carbon submicrospheric templates (nano-Au/C-template) functionalized by nisin

**Figure 2:**
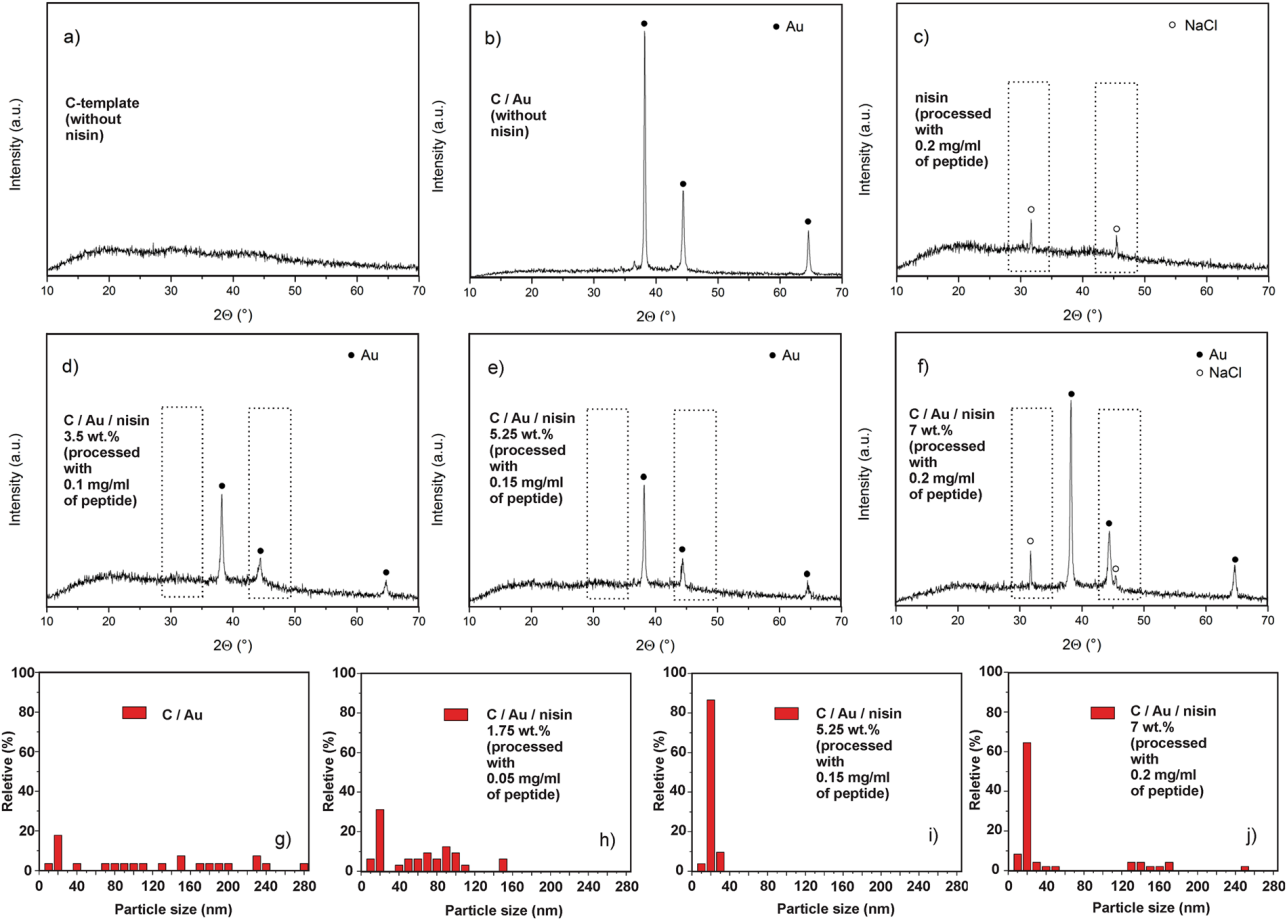
Composition and morphology determined by nisin. XRD patterns of *C* templates (**a**), *C* templates with Au nano-features (without nisin) (**b**), *C* templates with nisin (processed with 0.2 mg/ml of pure nisin) (**c**), *C* templates with Au nano-features functionalized with nisin (processed with 0.1, 0.15 and 0.2 mg/ml of pure nisin (**d**–**f**), respectively. Size distribution of Au nano-features on *C* templates (without nisin) (**g**), and Au nano-features on C templates processed with 0.1, 0.15 and 0.2 mg/ml of pure nisin (**g**–**j**), respectively.

The development of the technology for the formation of the template containing nano-features and bonding nisin to their surface has a few critical points. A carbon (C) template is formed using the hydrothermal decomposition of fructose, which yields an amorphous phase (Fig. 2a). After processing the template with the HAuCl_4_ precursor using a reflux approach, without the addition of the nisin, we obtained a C/Au composite (JCPDS no. 4-0784) (Fig. 2b) without the formation of any additional re-crystallized salts. It should be emphasized that the nisin used in this work was an unpurified peptide stabilized in NaCl and denatured milk solids. Therefore, the processing of nisin with the C template at a concentration of 8 ml/ml (with 0.2 mg/ml of pure nisin) provides re-crystallization of the salts from the precursor, which was verified by the presence of NaCl along the amorphous C phase (Fig. 2c). The same happened during the functionalization of Au with the highest concentration of unpurified nisin (8 mg/ml containing 0.2 mg/ml pure nisin). Along with the C/Au composite we detected a NaCl phase (Fig. 2f). Processing with lower concentrations (4 and 6 mg/ml with 0.1 and 0.15 mg/ml of pure nisin) did not provide any detectable NaCl (Fig. 2d,e).

During the optimization of the functionalization process we observed that nisin significantly affects the morphological characteristics of the Au phase. For instance, the thermally reduced Au phase, obtained without any functionalization with nisin, contained spherical and hexagonal particles with a wide size distribution ranging from a few up to 300 nm (Fig. 2g). A small fraction of them were attached to the surface of the C submicrospheres, forming a composite, while the majority were present as a separated phase. However, during the functionalization with nisin, when nisin induced the reduction of the Au-precursor and the formation of Au particles, their morphology was strongly dependent on the nisin content (2h–j). At a lower concentration (processed with 0.05 mg/ml and containing 1.75 wt.% of pure nisin) the obtained Au particles were spheres up to 150 nm in size with a small fraction attached to the C template (Fig. 2h). A further increase in the nisin content (processed with 0.15 and 0.2 mg/ml and containing 5.25 and 7 wt.% of pure nisin, respectively) produced 20-nm Au nanoparticles that were uniformly distributed on the surface of the C submicrospheres (Fig. 2i,j) and the total amount of the functionalized Au nanoparticles was deposited on templating submicrospheres within the composite, without the formation of additional phases.

Further investigations of the functionalization were performed using infrared spectroscopy (ATR technique). The spectra were compared for nisin, C spheres (without functionalization) and C/Au/nisin (with functionalization) (Fig. S1). Nisin exhibits a broad maximum at 3270 cm^−1^, which is attributed to N-H vibrations. On the other hand, C spheres exhibit broad absorption maxima at 3400 cm^−1^, which is related to the O-H vibrations. In the case of the C/Au/nisin there is the maximum at 3320 cm^−1^, which is the superposition of N-H vibrations and O-H vibrations from these two components in the material. We also observed the strong absorption maxima at 1580 cm^−1^ in the spectrum of nisin, assigned to C-O vibrations (the strongest for all absorptions for proteins). In this range, the light absorption for C/Au/nisin is stronger than for the C spheres, which proves the superposition of C- and nisin-related peaks in the C/Au/nisin.

**Figure 3:**
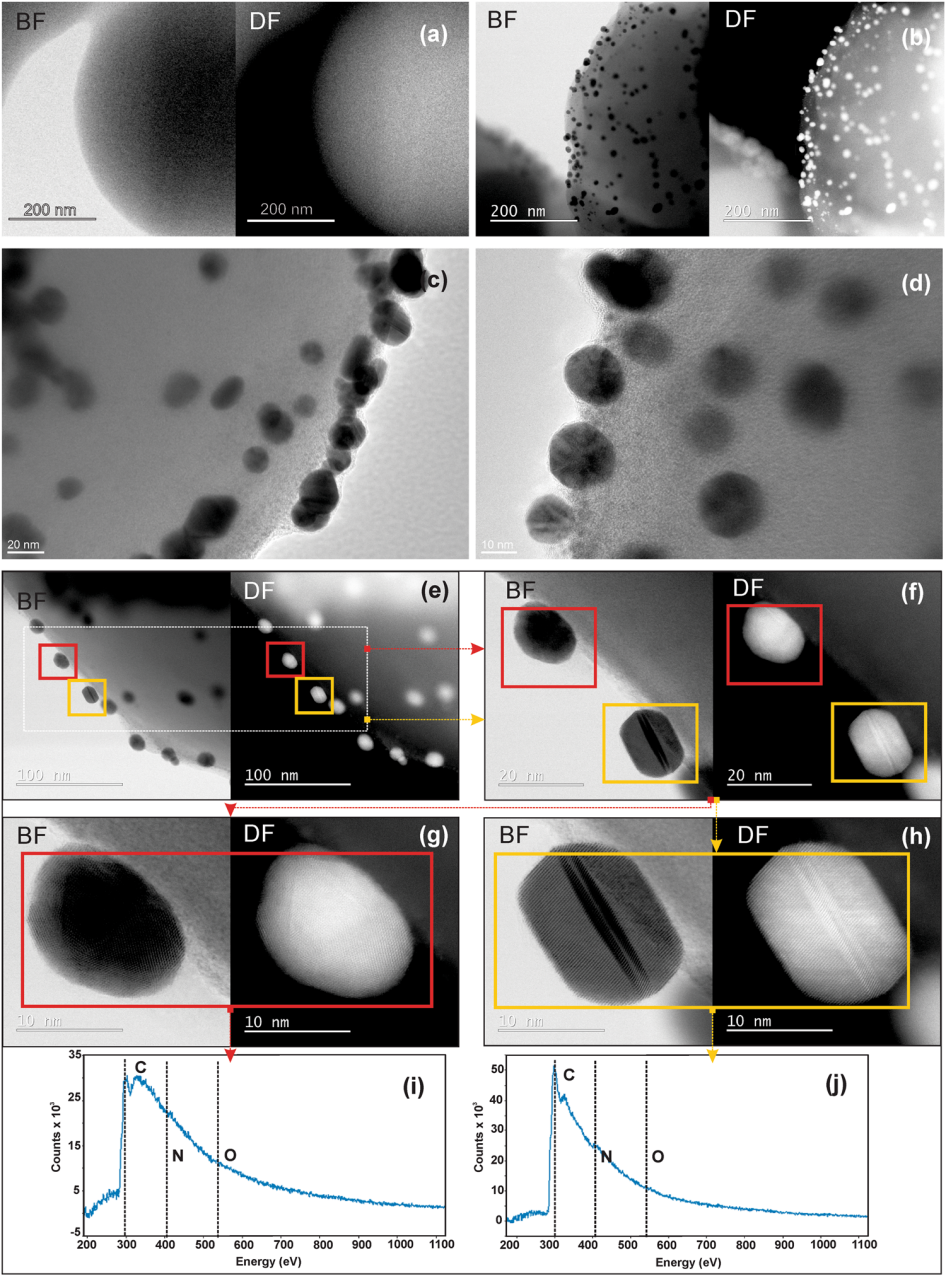
Functionalization of the surface of Au nano-features by nisin. Bright and dark filed (BF and DF) STEM images of *C*-template with nisin (without Au nanofeatures) (**a**) and *C*-template with nisin-functionalized Au nanofeatures (**b**); amorphous layer at the surface of nisin- functionalized Au nano-features at *C*-templates (**c**,**d**); BF and DF images of selected Au/nisin nano-features at the surface of *C*- templates (**e**,**f**) as well as selected spherical Au/nisin nano-features at the surface of *C*- templates (marked in red) (**g**) and selected twinned hexagonal Au/nisin nano-features at the surface of *C*- templates (marked in orange) (**h**) with EELS spectra taken from the surface of selected spherical (**i**) and selected twinned hexagonal (**j**) Au nano-features.

Processing the C template with nisin, without the presence of a Au precursor, did not result in the deposition of any additional nanoparticles on the surface of the C submicrospheres (Fig. 3a). On the other hand, when the Au phase was present, small Au nano-features were uniformly distributed on the surface of the templating C submicrospheres (Fig. 3b). The surfaces of these Au nano-features were covered by a thin amorphous layer (Fig. 3c,d), the elemental composition of which was checked in order to confirm the adsorption of the nisin (Fig. 3i,j). In addition to the glucose-sourced C-templating particles (rich in carbon and oxygen), nisin is characterized by a significant content of nitrogen-containing groups. The EELS spectra collected from the Au nanoparticles attached to a single C microsphere revealed core electron-energy-loss peaks corresponding to the C-K edge (284 eV), the N–K edge (401 eV) and the O-K edge (532 eV) confirming the presence of nisin. It was observed that Au particles attached to the surface of the C submicrospheres possess two different shapes - they are nanospheres and twinned nanohexagons (Fig. 3e–h). These two cases were isolated and an EELS analysis was performed on a single Au nanoparticle, showing spectra containing the N-K edge peak for both types of observed Au nanoparticles (Fig. 3i,j). Consequently, it was confirmed that the functionalization of the nisin on the composite takes place at the surface of the Au nano-features.

**Figure 4:**
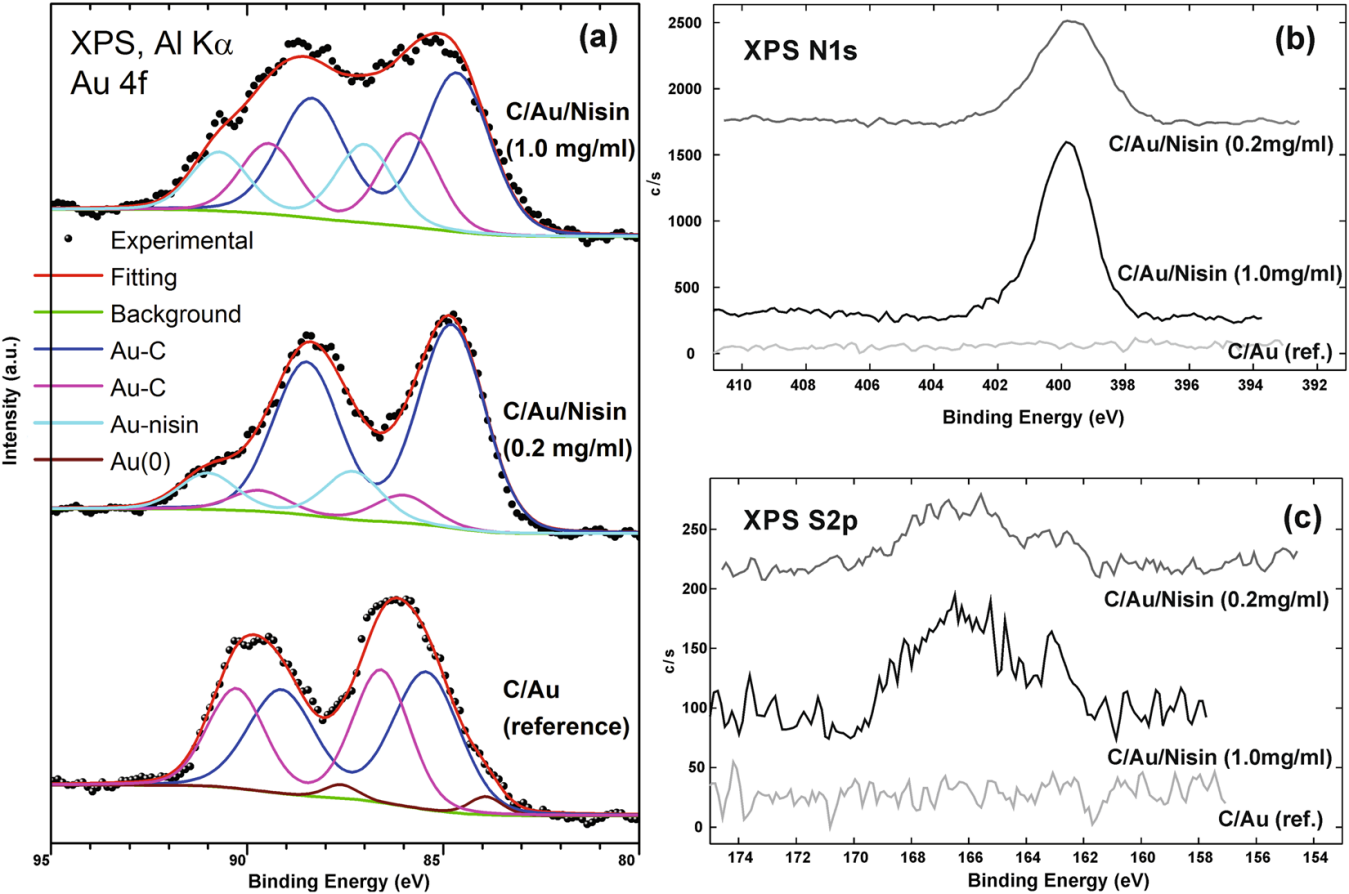
Elemental composition analysis at the surface of the C/Au. The Au 4 f (**a**), N 1 s (**b**) and S2p (**c**) spectra of the C/Au reference and C/Au/nisin functionalized with lower (0.2 mg/ml) and higher (1 mg/ml) content of the peptide.

Bonding nisin to the surface of Au nanoparticles deposited at C submicrospheres has been further investigated using X-ray photoelectron spectroscopy (XPS) (Fig. 4). For that purpose we investigated C/Au/nisin with lower (0.2 mg/ml) and higher (1 mg/ml) content of the peptide while C/Au (without functionalization) was selected as a reference. In comparison to the reference, both C/Au/nisin materials show presence of the nitrogen (Fig. 4b) and sulphur (Fig. 4c) with the intensities which increase with increased content of the peptide which additionally identified nisin bonded to the C/Au surface. Bonding of the peptide was further identified in Au 4f spectra. Functionalization of the C/Au with nisin resulted in shifting of the Au 4f maxima and their broadening with increasing the content of the peptide (Fig. 4a). Deconvolution of the Au4f maxima corresponding to the C/Au reference showed three components: one belonging to the metallic Au (at 83.8 eV) and two belonging to the Au bonded to C template (at 85.4–84.8 eV and at 86.5–85.8). In the case of C/Au/nisin deconvolution of Au4f revealed additional component (87.3–87 eV) with the intensity which increases with the content of the peptide and confirms binding nisin to Au.

Investigations of the surface properties, i.e., the zeta-potential and the specific surface area, gave important insights into the design of the C/Au/nisin. The C/Au spheres (without functionalization of the surface) possess a significantly negative surface with a mean value of the zeta-potential ζ_mean_ = (−32.1 ± 0.8) mV. In the case when nisin is added to the surface of the C/Au nanofeatures, the zeta-potential is changed and its mean value is ζ_mean_ = (−22.4 ± 0.6) mV. Consequently, we observed a decrease in the magnitude of the zeta-potential obtained after the addition of the nisin to the surface of the C/Au, which is assigned to the positive charge of the functionalizing, protein molecules. A determination of the specific surface area was performed for the C spheres (without nanofeatures on the surface) and C/Au/nisin (with functionalized nanofeatures). The results confirmed the low BET surface of the C spheres, which was 0.8 m^2^/g. After the addition of the nanofeatures the BET surface area of the C/Au/nisin was increased and it was 5.6 m^2^/g (7-times higher than for the C spheres). The charge and specific surface provided useful information for explaining the interactions of the C/Au/nisin with mammalian and bacterial cells.

Quantification of the nisin bonded to the surface of the C/Au revealed a very high efficacy, i.e., 89% of the initially used protein bonded to the surface of the Au nanofeatures. During the formation of the C/Au/nisin, the protein induces a reduction of the HAuCl_4_, induces the formation of Au nanoparticles and remains attached to their surface as positively charged functionalization.

### Susceptibility of nisin functionalized at nano-Au/C-template to Gram negative bacteria

**Figure 5:**
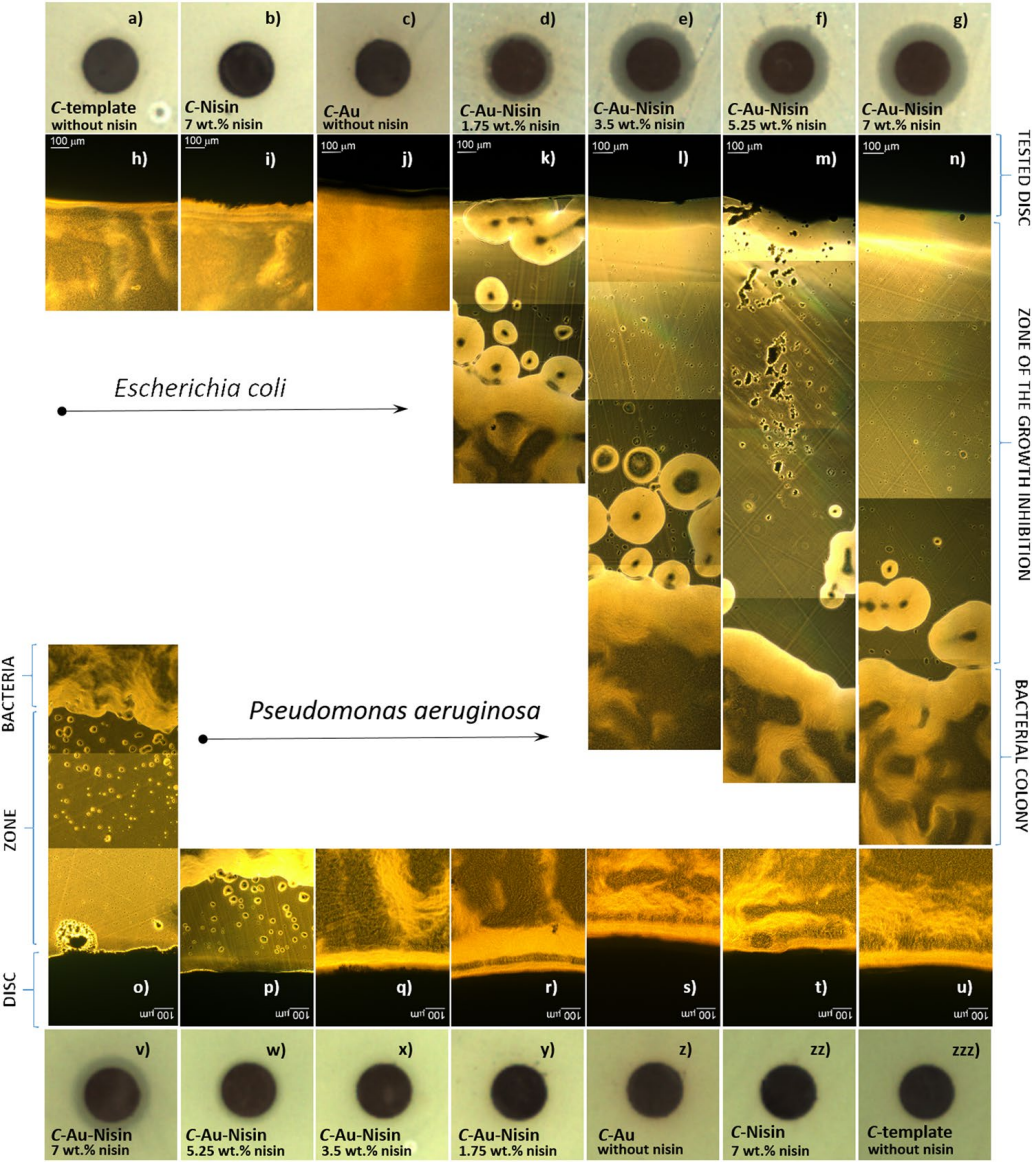
Susceptibility to Gram negative bacteria. Disc diffusion tests for the growth of: *Escherichia coli* around the pellets containing *C* -templates (**a**), *C*-templates/ nisin (containing 7 wt.% of pure nisin) (**b**), *C*-templates with Au nano-features (without nisin) (**c**) and *C*-templates with Au nano-features functionalized with nisin (containing 1.75, 3.5, 5.25 and 7 wt.% of pure nisin) (**e**–**g**) as well as *Pseudomonas aeruginosa* around the pellets containing *C* -templates (**zzz**), *C*-templates with nisin (8 mg/ml) (**zz**), *C*-templates with Au nano-features (without nisin) (**z**) and *C*-templates with Au nano-features functionalized with nisin (containing 1.75, 3.5, 5.25 and 7 wt.% of pure nisin) (**x–v**).

The developed composites were initially tested for susceptibility in two types of Gram negative bacteria, i.e., *E. coli* and *P. aeruginosa* (Fig. 5). The test included C/Au/nisin with different contents of the peptide (processed with 0.05–0.2 mg/ml of pure nisin and containing 1.75–7 wt.% of the peptide). The reference materials were C submicrospheres processed with and without nisin as well as C/Au obtained by thermal reduction without peptide. In the case of both types of bacteria, the references did not show any influence and the bacteria were able to grow close to the surface of the tested discs. On the other hand, the C/Au/nisin composite confirmed the susceptibility of both types of bacteria and showed an ability to induce a zone of inhibition for their growth. In the case of *E. coli* the susceptibility was higher and all four tested C/Au/nisin composites, containing 1.75–7 wt.% of the peptide, were able to inhibit the bacterial growth. The size of the zone was concentration dependent and its diameter increased with an increase in the nisin initially used for processing the composite. For *P. aeruginosa* the susceptibility was lower and among the four tested C/Au/nisin composites those processed with the highest concentrations (5.25 and 7 wt.% of pure nisin) confirmed the ability to form a zone of inhibition. Once again the susceptibility was concentration dependent and the diameter of the zone was increased with an increase of the initially applied nisin. A comparison of the susceptibilities of the composites with the tested bacteria revealed that the size of the growth-inhibition zone of the *E. coli* induced by the C/Au/nisin (with 1.75 wt.% of nisin) processed with the lowest concentration (0.015 mg/ml) of pure peptide was approximately the same as the size of the growth-inhibition zone of *P. aeruginsa* induced by the C/Au/nisin processed with the highest concentration of nisin (containing 7 wt.% of pure nisin). It should be pointed out that the susceptibilities of both types of bacteria to the C/Au-nisin composites were observed in spite of the presence of the NaCl phase, which is reported^14^ to have an inhibiting role in the antimicrobial activity of pure nisin.

In order to confirm the wide-spectrum antimicrobial activity of nisin within C/Au/nisin, the material was investigated for its susceptibility in a larger group of bacteria and stability (Fig. S2). This group included different Gram positive strains, including *Staphylococcus epidermidis* and some antibiotic-resistant Gram positive strains, like methicillin-resistant *Staphylococcus aureus* and vancomycin-resistant *Enterococcus faecium*. In contrast to the C/Au reference, C/Au/nisin (7 wt.%) confirmed its susceptibility in both *Staphylococcus* strains, while it was unable to affect the growth of VRE. Besides, the group included additional Gram negative strains, including *Salmonella enteritidis*, *Bacteroides fragilis* (anaerobe strain) and Amp-C *Enterobacter cloacae* (antibiotic-resistant Gram negative strain). In all three cases, C/Au/nisin confirmed its susceptibility, which was not observed for C/Au without nisin. Stability of the C/Au/nisin included susceptibility test for the freshly prepared material and material prepared 6 months ago. The susceptibility of the samples in *S. epidermidis* is exactly the same, which confirms the ability of the C/Au to retain the stability of the protein. This property is very important for future applications.

**Figure 6:**
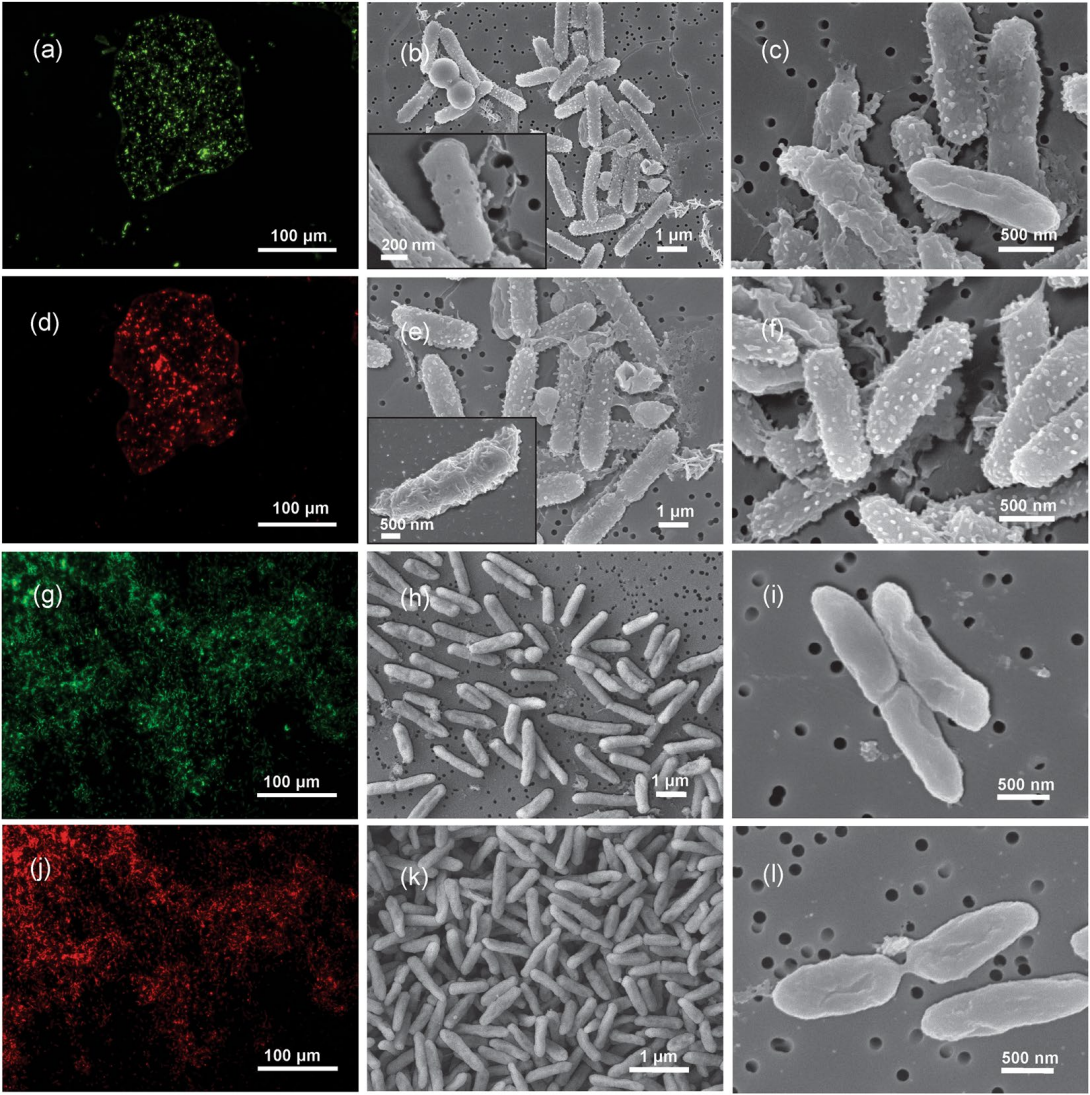
Disruption of the bacterial wall in Gram negative bacteria. Permeability of the membranes (SYTO/PI live/dead for fluorescence detection of dead/live bacteria (green/red)) in the case of *E. coli* (**a**,**d**) and *P. aeruginosa* (**g**,**j**) as well as morphological characteristics of the bacterial cells (SEM images) for *E. coli* (**b**,**c**,**e**,**f**) and *P. aeruginosa* (**h**,**i**,**k**,**l**) obtained after exposure to *C*-template with Au nano-features containing 7 wt.% of pure nisin.

The susceptibility in *E. coli* and *P. aeruginosa* was further confirmed during the live/dead test and the investigation of the morphological characteristics of the membrane obtained after the interactions with C/Au/nisin (7 wt.%). Both the *E. coli* and *P. aeruginosa* were exposed to the C/Au/nisin composite over a period of 24 hours. Afterwards, staining was performed using SYTO/PI fluorescent dyes that detect the permeability through the bacterial membrane. The SYTO is a permeable green fluorescent dye that crosses the cell-membrane and binds to the nucleic acids of both dead and viable cells. On the other hand, PI is a red fluorescent dye that is impermeable to intact membranes, penetrates only mechanically destroyed or damaged membranes and binds to the nucleic acids of dead bacteria^24^. The SYTO/PI staining of *E. coli* after exposure to C/Au/nisin (7 wt.%) showed intense green/red fluorescence and confirmed that the majority of the bacteria had damaged membranes (Fig. 6a,d). A similar situation was observed after staining *P. aeruginosa* exposed to C/Au/nisin (7 wt.%), which revealed an intense green couple with an intense red fluorescence, confirming that the majority of the bacteria had damaged membranes (Fig. 6g,j).

The morphology of the *E. coli* obtained after exposure to the C/Au/nisin (7 wt.%) revealed significant modifications (Fig. 6b,c,e,f). These modifications were observed in all the detected bacterial cells. The majority of the bacteria showed an altered texture of the outer membrane. Instead of the smooth surface characteristic for the intact bacterial cells, their surface became sphere-like and with fibrous features. In some cases we observed the formation of small punctures in the surfaces of the cells, while other bacterial cells possessed large perforations and the whole structure of their cell was disintegrated and destroyed. On the other hand, for *P. aeruginosa* the majority of the observed bacteria showed an intact structure of the cells with a smooth surface. In a small fraction of them we observed large perforations of the membrane; however, changes in the roughness that were detected in the *E. coli* were not observed in the *P. aeruginosa*.

In the next step the antibacterial activity of C/Au/nisin in *E. coli*, *P. aureus* and *S. epidermidis* was investigated for the bactericidal effect (Fig. S3). C/Au/nisin (7 wt.% of nisin), together with the references corresponding to the bacteria without materials and bacteria exposed to C/Au (without nisin), were incubated with bacteria at concentrations of 10^8^ CFU/ml and 10^6^ CFU/ml for different periods of time. Afterwards, they were tested for their ability to undergo further growth after an additional 24 hours of incubation. Except for the C/Au/nisin (7 wt%) (for all three types of bacteria) all of the bacteria exposed to C/Au proceeded with growth. In the case of exposure to C/Au/nisin (7 wt.%), any further growth was completely prevented, which confirmed the bactericidal effect of the material. It was interesting to observe that the bactericidal effect of C/Au/nisin for the concentration of 10^6^ CFU/ml of bacteria was obtained in 3 hours, while for 10^8^ CFU/ml, the bactericidal effect was obtained after 8 hours.

**Figure 7:**
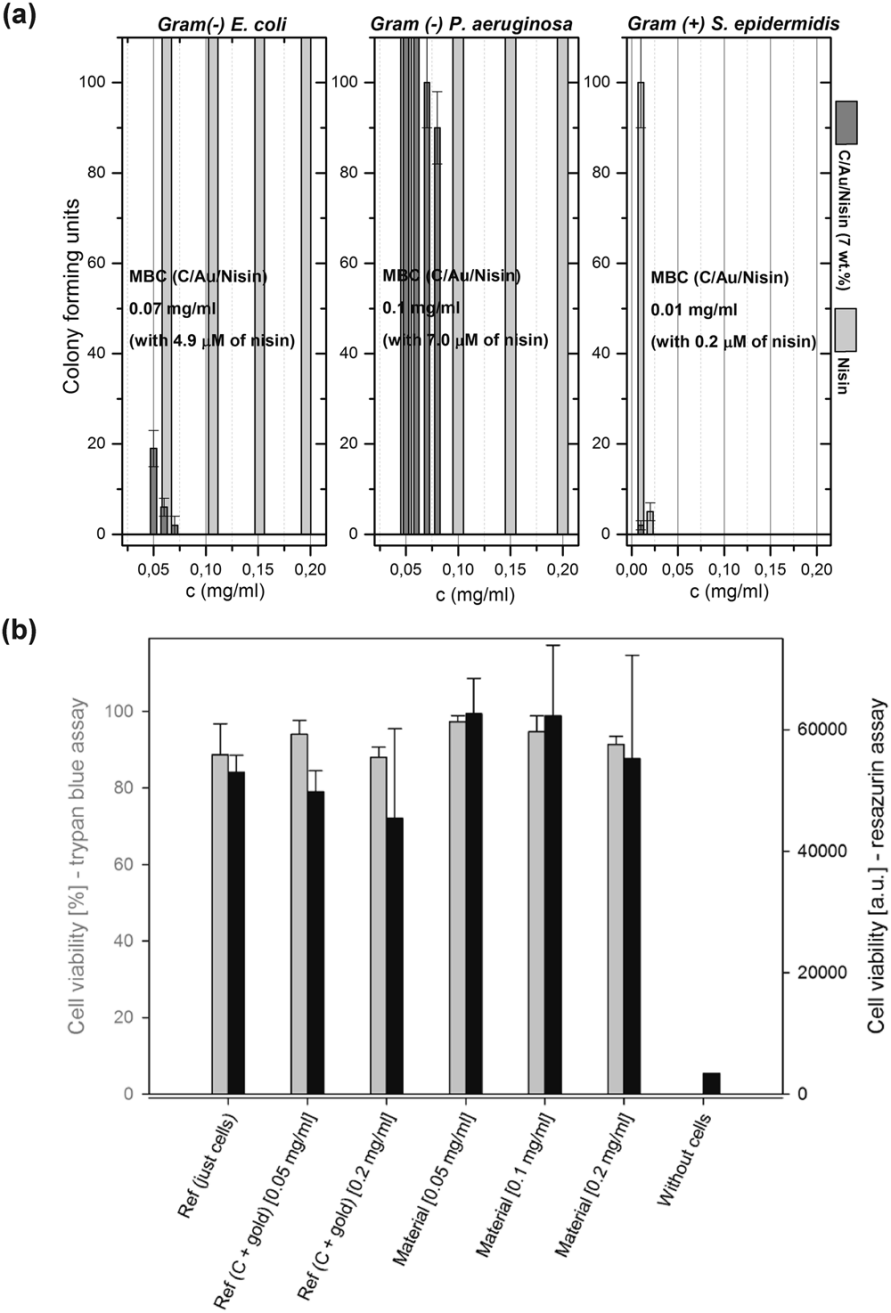
Minimal bactericidal concentrations (MBC) and viability of mammalian cells. MBC concentrations determined for *E. coli* and *P. aeruginosa* as Gram negative strains and *S. epidermidis* as Gram positive strain (**a**); viability of L929 fibroblasts exposed to different concentrations of C/Au/nisin (containing 7 wt.% of pure nisin) determined by Trypan blue (grey) and Resazurin assay (black). The error bars present the standard deviation of three measurements.

Minimal bactericidal concentrations (MBC) were determined for *E. coli*, *P. aeruginosa* and *S. epidermidis* (Fig. 7a). For the both Gram negative strains we noticed very similar activity. The lowest concentration of the material capable to kill *E coli* was 0.07 mg/ml while for the *P. aeruginosa* it was 0.10 mg/ml (Fig. S4). The material contains 7 wt% of nisin meaning that 4.9 μg/ml (1.5 μM) and 7 μg/ml (2.0 μM) of the protein provided fast bactericidal activity (after 3-hour exposure). For the tested concentrations nisin, provided as a reference, was not able to kill Gram negative bacteria with the same kinetics as nisin-functionalized C/Au.

In the case of Gram positive bacteria, *S. epidermidis*, (Fig. S5) the activity was much stronger and it was detected for both nisin-functionalized C/Au and nisin-reference. We observed that the lowest concentration of the C/Au/nisin capable to kill bacteria was 0.01 mg/ml. This material contains 7 wt% of nisin meaning that 0.7 μg/ml (200 nM) of the protein provided fast bactericidal activity (after 3-hour exposure). Nisin, provided as a reference, was able to kill bacteria after short 3-hour exposure for the concentrations of 0.02 mg/ml and higher.

### Viability of nisin functionalized at nano-Au/C-template

Interactions between Au/C/nisin and mammalian cells were tested in L929 fibroblasts using two approaches: a Trypan blue assay, which measures the compactness of the cellular membrane, and a Resazurin assay, which measures the mitochondrial activity. After exposure to the different concentrations of materials (C/Au and C/Au/nisin (7 wt.%)) the cells did not show any morphological changes that could be assigned to the first signs of toxicity (Fig. S6). In the case of the higher concentration of C/Au (0.2 mg/ml), without nisin, we observed a decrease in the total number of cells; however, further quantification (using Trypan blue) revealed that the majority of the detected cells remained viable. It was also the case for the cells exposed to different concentrations of C/Au/nisin (7 wt.%), and the obtained viability was comparable to the viability of the cells grown without material. Investigations based on metabolic activity using the Resazurin assay showed comparable results. After exposure to the different concentrations of C/Au and C/Au/nisin (7 wt.%) the staining confirmed the same change of the colouring as for the viable cells grown without materials. The fluorescence spectra of the dyes with materials (without cells) showed very low counts and excluded interferences of the material with the measurements. Quantification of the results confirmed the high level of the viability, with observed differences in the case of the materials with and without nisin (Fig. 7b). For the C/Au/nisin (7 wt.%) the viability of the cells was generally higher than for the C/Au. More detailed investigations are needed to reveal the exact reasons, which could be related to the positively charged C terminus of the nisin with a higher affinity for interactions with bacterial cells. As previously shown, for the 0.2 mg/ml of C/Au/nisin (7 wt.%), it was possible to induce the bactericidal effect in *E. coli*, *S. epidermidis* and *P. aeruginosa*, the viability of the L929 fibroblasts was higher than 90%, which is comparable to the viability of the reference cells grown without materials.

## Discussion

An intensive study, conducted in order to identify the mechanism of natural antibacterial activity of nisin against Gram positive bacteria, confirmed the ability of this protein for the complexation of lipid II molecules in the surface peptidoglycan layer. The complexation takes place between extracellular-oriented pyrophosphate groups of lipid II and the *N-*terminal end of nisin^25^. Specific bonding of the nisin to lipid II through the formation of the stable hydrogen bonds^26^ affects the synthesis of the cell wall, resulting in dead Gram positive bacteria. After bonding to lipid II (as a docking molecule) the *C* terminal can cross the membrane directly, participating in the formation of the pore^27^. Since lipid II molecules are not abundant in peptidoglycan (less than 1 mol% of membrane phospholipids) the specificity of nisin to pyrophosphates in the huge amount of other phospholipids was not clear^28^. Recent investigations suggested the existence of the “amphiphilic pattern” formed of hydrophilic and hydrophobic domains created by the single lipid II molecule surrounded by the rest of the membrane. This pattern is believed to be the reason for the high affinity recognition of the lipid II by nisin^28^.

Gram negative bacteria possess a characteristic cell wall composed of an outer and inner (cytoplasmic) membrane connected by a thin peptidoglycan layer^2^. The outer membrane is mainly composed of glycolipids (lipopolysaccharides, LPS) that form 75% of their surface^29^. Due to the phosphate and carboxylate groups in sugar acids the cell surface is negatively charged^30^. Since nisin specifically binds to lipid II in peptidoglycan it is only efficient against Gram positive bacteria, while Gram negative bacteria are protected by the outer membrane^2^. However, as we showed, the functionalization of nisin at the surface of the C/Au nanocomposite ensures the formation of the biomaterial that produces activity against different Gram negative bacteria, including antibiotic-resistant species.

Based on the molecular dynamics simulations and experimental evidence, a very recent study^31^ confirmed that high concentrations of nisin are able deform the bacterial cell membrane, even in the absence of lipid II molecules. The authors proposed non-specific physico-chemical interactions of nisin with phospholipids. The mechanism enables the deformation of the membranes and the generation of pores at the surface of both the Gram positive and Gram negative bacteria induced by the membrane tension caused by the high surface bound density of nisin. Since it is driven by the steric volume effect in solution, in the case of the freely solubilized nisin molecules investigated in this study, the mechanism is valid only for very high concentrations (i.e., the MIC for *E. coli* was determined to be 12 μM).

The C/Au/nisin composite presented in this work possesses a specific architecture made of C submicrospheres used as a template for the uniform deposition of the small Au nanoparticles with the surface functionalized by nisin. As we observed, after the formation of the Au (by C-template-induced reduction of the precursor), peptide attaches to the surface of the nanoparticles and remains bonded as their functionalization. Consequently, there is a re-distribution of the charge, and the surface of the material obtains a positive potential. Moreover, the nanofeatures assembled at the surface of the C spheres significantly increase the specific surface area of the material; the architecture of the composite prevents aggregation and provides a large contact area between the nisin and the membrane of the Gram negative and Gram positive bacteria. With this structure, C/Au/nisin does not use only the natural antibacterial mechanism of the nisin, but rather participates in the new mechanism, determined by the novel structure, which is based on a contact with the surface of the bacterial cells. The activity is a consequence of the intense electrostatic interactions that occur between the highly positively charged surface of the nanocomposite and the negatively charged groups in the outer membrane of the Gram negative bacteria. Similar to the recent study^31^, these interactions are intense enough to provide a disintegration of the membrane and enable the antimicrobial activity of nisin in both Gram positive and Gram negative bacteria. However, in contrast to the previous study, when activity was tested for freely solubilized nisin molecules and proven to be valid only for high concentrations^31^, we showed that the same effect can be achieved for significantly lower concentrations of the nisin by the process of functionalization. Functionalization provides a locally high density of the nisin molecules available for interactions with membranes, which creates a tension able to disintegrate their structure. Another step ahead in comparison to the previous study is testing the proposed non-selective physico-chemical mechanism^31^ in mammalian cells and an investigation of its influence on their membranes. As we showed, the mechanism does not have the same effect on mammalian cells and they retain their compactness (and remain viable at high concentration), which may be associated with completely different surface potential and positive surface charge in mammalian cells.

Bonding nisin to Au nano-features assembled on the surface of *C* templates can take place by attaching terminal (*C*- or *N*-terminal) or side groups (i.e., NH_3_- groups in lysine residues or -S- groups in thioether rings). Bonding to side groups might be prohibited by steric interactions and would probably alter the structure of the peptide. Since nisin bonded to the Au/C shows very similar CD characteristics (CD spectra in Fig. S8) as the pure peptide, the functionalization did not affect the structure of the initial peptide. For that reason our opinion is that bonding nisin to Au nano-features using side groups is unlikely. Therefore, the most probable option is that nisin could be attached to Au using terminals. The high affinity of gold to bond N-containing groups^12^ and the hydrophobic nature of nisin’s *N*-terminal^32^, which is more prone to interact with hydrophobic Au, are the reasons we believe that bonding takes place through the *N*-terminal end, while the *C*-terminus (which holds the most of the positive charge of the peptide- i.e. Lys- 22, His-27, His-31, Lys-34)^12^ remains free for interactions with bacterial membranes. However, additional investigations and more detailed study are needed to confirm exactly the orientation of the nisin bonded to Au nanoparticles within C/Au composite.

It should be highlighted that in comparison with other strategies used to obtain the activity of nisin with respect to Gram negative bacteria, when the outer membrane is pre-treated to be disintegrated^14^, the C/Au/nisin nanocomposite is capable of providing disintegration by itself, without additional treatment. Moreover, previous studies showed the need for purification of the nisin and removal of the salts and milk residues, since they decrease the permeability of the peptide through the membrane and prevent activity against Gram negative bacteria^15, 16^. As experimentally evidenced by K. Kuwano *et al*.^16^, purified nisin is effective against both *E. coli* and *S. aureus*. However, the addition of salts (i.e., NaCl) inhibits both activities. Accordingly, they proposed a salt-sensitive mechanism of action in nisin. Based on this, it is possible to think about inhibition of the nisin’s activity in the organism or about the accumulation of salts as a tool for bacteria to develop resistance. We wanted to point out that the strategy we developed is not sensitive to the salts (NaCl) and that nisin attached to the surface of C/Au nanofeatures retains its activity, even in the case when salts are present. The formation of the conjugate between the nisin and the Au is proven to increase the stability of the peptide and prevent its inactivation by a variation of the pH^33^. As we showed, the mechanism provides activity besides the presence of NaCl in the system.

On the other hand, different approaches (covalent grafting^22, 23^, co-encapsulation^20^, molecular cloning^21^, etc.) presented in the literature as strategies for broadening the activity of nisin are based on a synergy of the activity of nisin against Gram positive bacteria with the activity of another antimicrobial agent active against Gram negative bacteria (Ag nanoparticles^20^, other peptides, i.e., apidaecin 1b, oncocin^21^). In contrast, the approach presented in this work is focused on a structural modification of nisin at the surface of Au nanofeatures, assembled at the surface of the C templating spheres, which extends the activity particularly related to this peptide to a wide range of different Gram positive/negative, aerobe/anaerobe and antibiotic, multi-resistant bacterial strains.

Recent investigations performed on exploring antimicrobial peptides for designing new therapeutic approaches have been focused on virus-sourced antimicrobial peptides^34^ and their application as membrane-penetrating molecules for cell-specific drug delivery^35^. The major advantages of these approaches are associated with their capacity for intensive interactions, selectivity and transportability through the cellular membranes. This context can be very effectively extended by the application of the nano-engineering approach and using Au nanoparticles functionalized by lantibiotics (i.e., nisin). A combination of the confirmed safety of these types of peptides with a virus-like capacity to target membranes are their major advantages, which will be very interesting to explore in the future for designing post-antibiotic drugs.

In conclusion, the attachment of nisin, as a cationic peptide with a highly positive charge, onto the surface of a C/Au nanocomposite is an innovative strategy for obtaining the antibacterial activity of this peptide against Gram negative bacteria. The nanostructure used as a carrier of the nisin enables an effective interaction of the peptide with the bacterial membrane that uses intensive electrostatic interactions for the disintegration of its structure and provides antimicrobial activity. The strategy is very effective since it does not require additional pre-treatment of the bacteria or additional purification of the nisin in order to provide activity against Gram negative bacteria, which is its major advantage in comparison to currently applied technologies. Broadening the activity of nisin to Gram negative bacteria is very important from the perspective of extending the application of this peptide from the sector of food preservation to the sector of healthcare. The strategy carries the potential for designing a next generation of efficient, safe, economic and biologically-sourced, non-antibiotic antimicrobials.

## Methods

### Synthesis of C submicrosphere templates

Carbon submicrospheres were prepared hydrothermally in a Teflon-lined autoclave by treating a 0.625- M aqueous solution of fructose at 150 °C for 6 h. The obtained black product was isolated by filtration and then purified by centrifugation/washing and re-dispersed in an ethanol/water solution five times. Afterwards, the carbon spheres were air-dried at ambient temperature for 24 h. The as-prepared C templates were used as supports for the nisin-functionalized Au nanoparticles.

### Formation of the functionalized Au deposited on the C – submicrosphere templates

Some 50 mg of C submicrosphere templates were dispersed in 50 ml of distilled water (ρ > 18 MΩ) and heated under reflux. After reaching a temperature of 50 °C, 25 ml (1 mg/ml) of chloroauric acid (HAuCl_4_ × H_2_O; 99.9%; Au 49% min; Sigma Aldrich) water solution and 25 ml of nisin (from *Lactococcus lactis*; 2.5% in balance NaCl and denaturized milk solids, Sigma Aldrich) solution were added and synthesized for 3 h. The solutions used contained 0.05 mg/ml, 0.1 mg/ml, 0.15 mg/ml and 0.2 mg/ml of pure nisin. Afterwards, the suspension was centrifuged two-times (6500 rpm for 30 min) to separate the particles from the supernatant and air-dried on a watch glass.

### Characterization techniques

#### Physicochemical characteristics

Phase-composition analysis was performed on an x-ray diffractometer D4 Endeavor AXS (Bruker, Billerica, MA) equipped with a Cu-K_α_ x-ray source. The spectra were collected in the 2θ range between 10 and 70° with a step size of 0.04° and 3 s collection time. Morphological characterization was performed at 200 kV on a transmission electron microscope (JEM-2100, JEOL Inc., Tokyo, Japan) equipped with a slow*-*scan CCD camera ORIUS SC1000A (Gatan Ltd.). A stereological analysis was performed on 500–700 particles using an image analyzer (Image Tool Win 3.0 Software). The structure and chemistry of the functionalized and deposited composites were examined with a JEOL ARM 200 F CF scanning- transmission electron microscope with atomic resolution, equipped with a cold field-emission gun, a probe spherical aberration corrector (CESCOR unit from CEOS, Germany) and an energy filter (QuantumGIF, Gatan, USA) for electron energy-loss spectroscopy with DualEELS capability. The chemistry of the surface and functionalization by the protein were additionally confirmed using infrared spectroscopy (ATR technique). The analysis was conducted on a Perkin Elmer Spectrum 400 MIR spectrophotometer using the attenuated total reflection (ATR) technique. The specific-surface-area measurements were performed using the Brunauer-Emmett-Teller (BET) method with a Micromeritics Gemini II 2370 nitrogen-adsorption apparatus (Norcross, GA). Zeta-potential measurements were performed using a Pals Zeta Potential Analyzer 5.71 (Brookhaven Instruments) in water (pH = 5) using a concentration of 0.2 mg/ml of the tested material. Elemental analysis on the surface was performed using PHI-TFA XPS spectrometer equipped with Al-monochromatic source. All of the data were obtained by averaging at least three repeated integrations of the peaks.

#### Protein quantitative analysis

The supernatant, obtained after synthesis of the C/Au/nisin, was centrifuged at 18000 g for 30 minutes, to remove residual particles and to obtain a clear transparent solution containing residual, non-bonded protein. Aliquots of 100 μl were transferred to the microtiter plate and the Bradford method for determining the proteins was used for the amount of nisin in the supernatant. A 20 μl of concentrated Coomassie dye reagent and 80 μl of physiological solution were added to the sample aliquots and absorption was measured at 595 nm. The concentration of the remained nisin was determined using the calibration curve and used to calculate the concentration of the protein within C/Au/nisin material.

#### Disc diffusion test

The susceptibility of the composites was tested against the following bacterial strains: methicillin-resistant *Staphylococcus aureus* (MRSA) (ATCC 43300), *Staphylococcus epidermidis* (ATCC NCIMB8853) and vancomycin-resistant *Enterococcus faecium* (ATCC 51299) as Gram positive strains as well as in *Salmonella enteritidis* (ATCC 14028), *Bacteroides fragilis* (ATCC 23745), Amp-C *Enterobacter cloacae* (NEQAS 1719), *Escherichia coli* (MG1655) and *Pseudomonas aeruginosa* (MW1) as Gram negative strains, using the disc diffusion test, known as the Kirby-Bauer method. The model bacteria strains were cultured by 16 h of incubation in a Müller-Hinton broth and collected during the logarithm phase of growth. The bacteria suspension was diluted to 10^6^ CFU/ml and covered on agar plates (Φ = 90 mm). The tested materials were pressed into 6-mm discs (containing 15 mg of the material), placed on an agar plate covered with the model bacteria and incubated at 37 °C for 24 h. After the incubation, the zone of inhibition of the bacteria growth was measured, photographed and observed with an optical microscope. All the tests were performed twice.

#### Bactericidal test

The 2 ml of bacteria (*S. epidermidis*, *E. coli* or *P. aeruginosa*) (10^6^ CFU/ml and 10^8^ CFU/ml) in the phosphate buffer were exposed to C/Au/nisin (0.2 mg/ml of the material formed using 0.2 mg/ml of pure nisin) for different periods of time. The negative control were bacteria grown without material. Bacteria exposed and non-exposed to material were transferred to agar plates and incubated for an additional 24 hours to test the bactericidal influence.

#### Live/Dead bacteria test

After the incubation of bacteria (*E. coli*, *S. epidermidis* or *P. aeruginosa*) for the bactericidal test (using 0.2 mg/ml of the material processes with 0.2 mg/ml of pure nisin and containing 7 wt.% of peptide), suspensions were centrifuged at 2000 g for 10 minutes and the supernatant was poured out. Two hundred µL of fresh phosphate buffer was added, the pellet was re-suspended and mixed with the Live/Dead BackLight kit components. After 15 minutes of incubation, the green- and red-light fluorescence were detected using Olympus IX81 inverted research microscope.

#### SEM analysis of bacteria

The morphological properties of the *E. coli* and *P. aeruginosa* were assessed for bacteria prepared according the macro-dilution procedure previously described for Live/Dead testing. The bacteria were fixed in 1% formaldehyde and 0.5% glutaraldehyde in a 0.1- M phosphate buffer solution, post-fixed using a 1% aqueous solution of osmium-tetroxide for 1 hour, dehydrated in 10%, 30%, 50%, 70%, 90% and 100% ethanol solutions and dried using the critical-point drying method in a CPD030 dryer (Balzers). Before the SEM examination the samples were coated with a 3-nm layer of platinum in a sputter-coater SCD 050 (BAL-TEC).

#### Viability tests

The test was performed for L929 osteoblast cell line. C/Au/nisin (7 wt.% of nisin) was dispersed using sonication in a cell-growth medium in concentrations of 0.05 mg/ml, 0.1 mg/ml and 0.2 mg/ml. Prepared suspensions were added on the cultured cells in 96-well plates (approximate 80–90% confluence) in sextuplicate and incubated for the 24 hours.

#### Trypan blue assay

After 24-hour incubation of cells with C/Au/nisin, cell-growth media were removed from the seeded 96-well plates. Phosphate buffer saline (PBS) was rinsed three times to remove all the serum proteins remaining in the wells for effective cell dissociation from the culture surface using trypsin. After trypsinization cells were re-suspended with cell-growth media and put on a centrifuge (Hettlich-Micro 22 R, 1800 rpm, 3 min). After removing the supernatant the cell pellet was re-suspended with 50 µl PBS followed by the addition of Trypan blue stock solution (0.4% (w/v), Thermo Fisher Scientific) in a volume ratio 1:1. Total volume of suspension (100 µl) with the cell number of approximately 20000 was incubated in Trypan blue for 2 min and then transferred under the hemacytometer to measure the cell viability. Parallel measurement of cell viability using Trypan blue test was done on the cultured cells, where Trypan blue solution was incubated with cells for 3 min.

#### Resazurin assay

Cell viability was further tested using fluorometric analysis by Resazurin (Invitrogen) assay. Resazurin was diluted in cell-growth media in a concentration of c = 560 µM. 200 µl solution was added on the cultured cells (incubated with the investigated material) in 96-well plates and left incubating for 4 h. After 4 h the solution was removed from the wells and tested on a fluorescence microplate reader (Tecan, Infinite M1000). Resazurin is irreversibly reduced to the pink-colored and highly red fluorescent resorufin during oxidation-reduction processes in cell mitochondria, indicating the mitochondrial function and thus cell viability. The emission fluorescence spectrum of resorufin was recorded using λ = 560 nm excitation light. The spectral peak intensity at approximately λ = 560 nm directly indicates the cell viability.

## Electronic supplementary material

Supplementary information
