# Supplementary material for: Nano-engineering the Antimicrobial Spectrum of Lantibiotics: Activity of Nisin against Gram Negative Bacteria
Source: Sci Rep. 2017 Jun 28;7:4324. doi: 10.1038/s41598-017-04670-0 (PMC5489483; doi:10.1038/s41598-017-04670-0)
Supplement: Supplementary file 1 — Supplementary information [file 41598_2017_4670_MOESM1_ESM.pdf]

Supporting Information

Nano-engineering the Antimicrobial Spectrum of Lantibiotics: Activity of Nisin against Gram Negative Bacteria

Marija Vukomanović,[1,*] Vojka Žunič,[1] Špela Kunej,[1] Boštjan Jančar,[1] Samo Jeverica,[2] Rok Podlipec,[3] Danilo Suvorov[1]

[1]Advanced Materials Department, Jožef Stefan Institute, Jamova 39, 1000 Ljubljana, Slovenia

[2] Institute for Microbiology and Immunology, Medical Faculty, University of Ljubljana, Zaloška 4, 1000 Ljubljana, Slovenia

[3] Laboratory of Biophysics, Condensed Matter Physics Department, Jožef Stefan Institute, Jamova 39, 1000 Ljubljana, Slovenia

* marija.vukomanovic@ijs.si , phone: +386 1 477 35 47, fax: +386 1 251 9385.

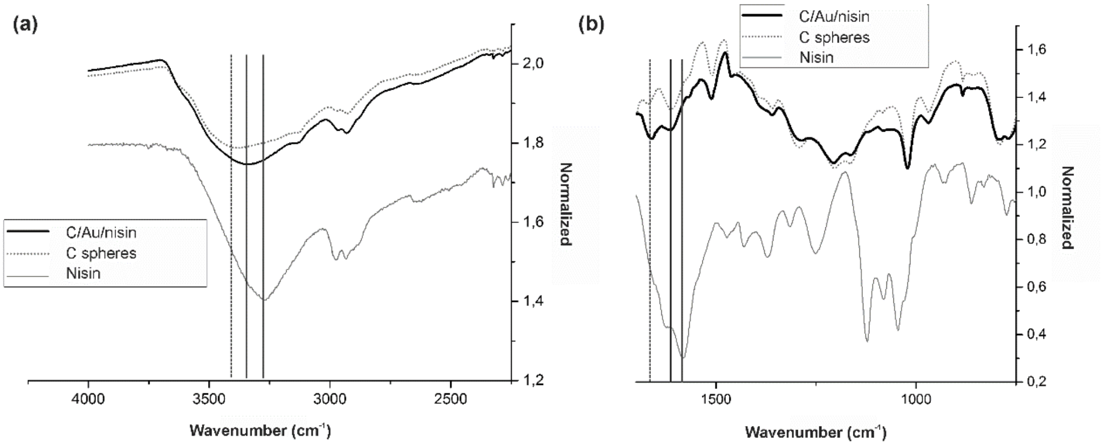

Fig. S1: Comparative ATR spectra of nisin, *C*-templating spheres without nisin and C/Au/nisin material containing Au nano-features on C-templating spheres functionalized with nisin (processes with 0.2 mg/ml and containing 7 wt.% of pure nisin).

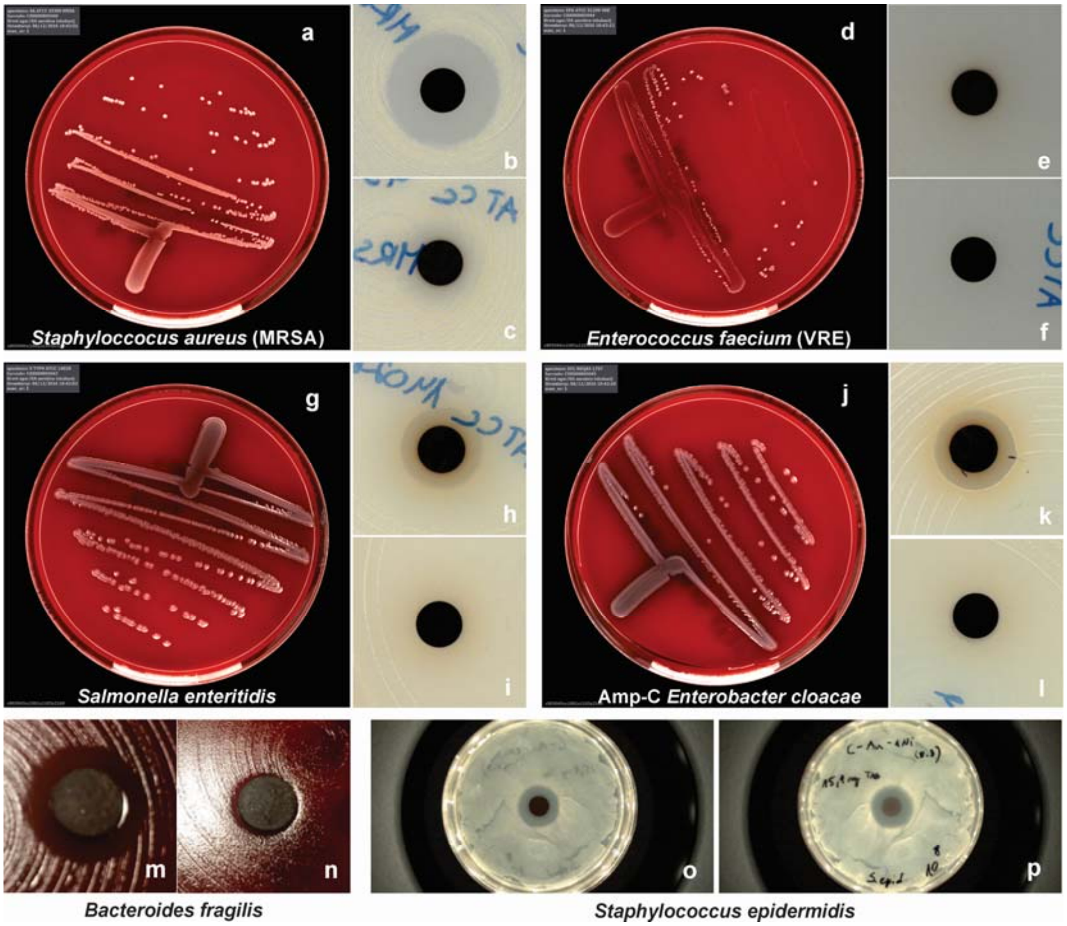

Fig. S2: Figure 5. Susceptibility tests for C/Au/nisin (containing 7 wt.% of pure nisin) and C (used as control) in different Gram(+) and Gram(-) bacterial strains: methicillin-resistant Staphylococcus aureus

(a- bacteria, b- with nisin, c- without nisin), vancomycin-resistant Enterococcus faecium (d- bacteria, e- with nisin, f- without nisin), Salmonella enteritidis (g- bacteria, h- with nisin, i- without nisin), Amp-C Enterobacter cloacae (j- bacteria, k- with nisin, l- without nisin), Bacteroides fragilis (m- with nisin, n- without nisin) and Staphylococcus epidermidis (o- freshly-prepared and p- 6-months old composite).

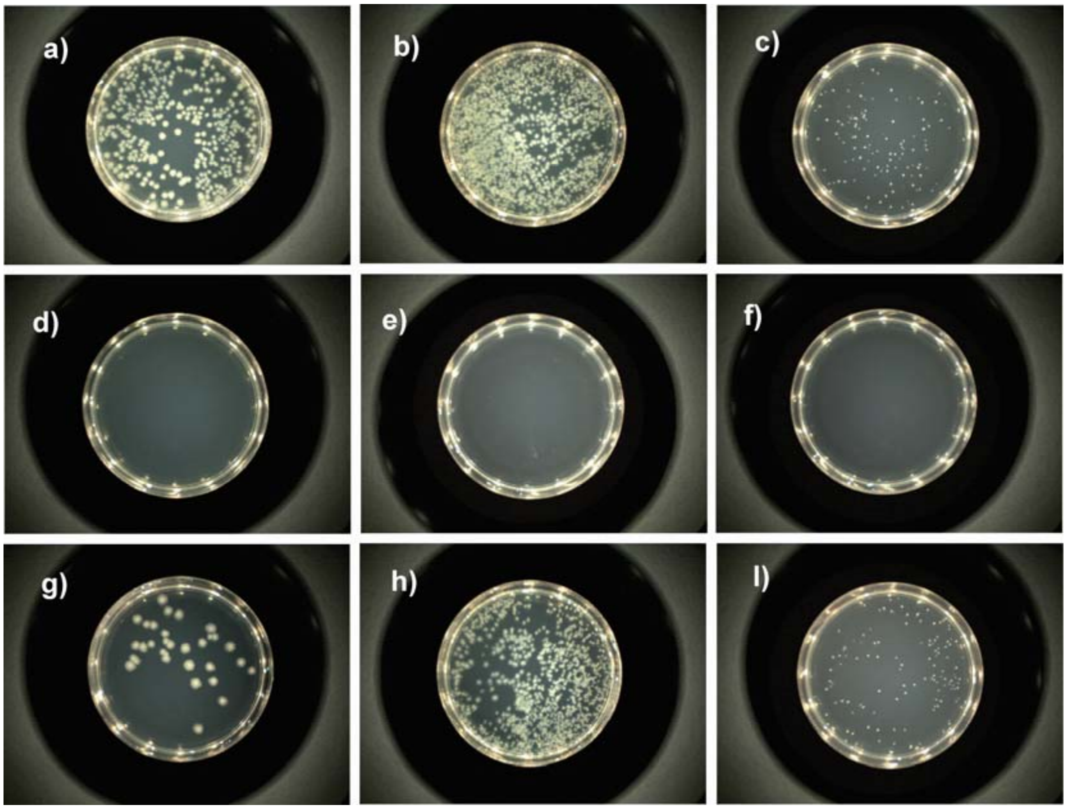

Fig. S3: Bactericidal test. Normal growth of the bacteria (a,b,c), completed inhibition of the growth during 24-hour incubation of bacteria previously exposed to 0.2 mg/ml of C/Au/nisin (containing 7 wt.% of pure nisin) during 8 hours (d,e,f) and partial growth of the bacteria exposed to C/Au (without nisin) (h,I,j) for E. coli, P. aeruginosa and S. epidermidis respectively.

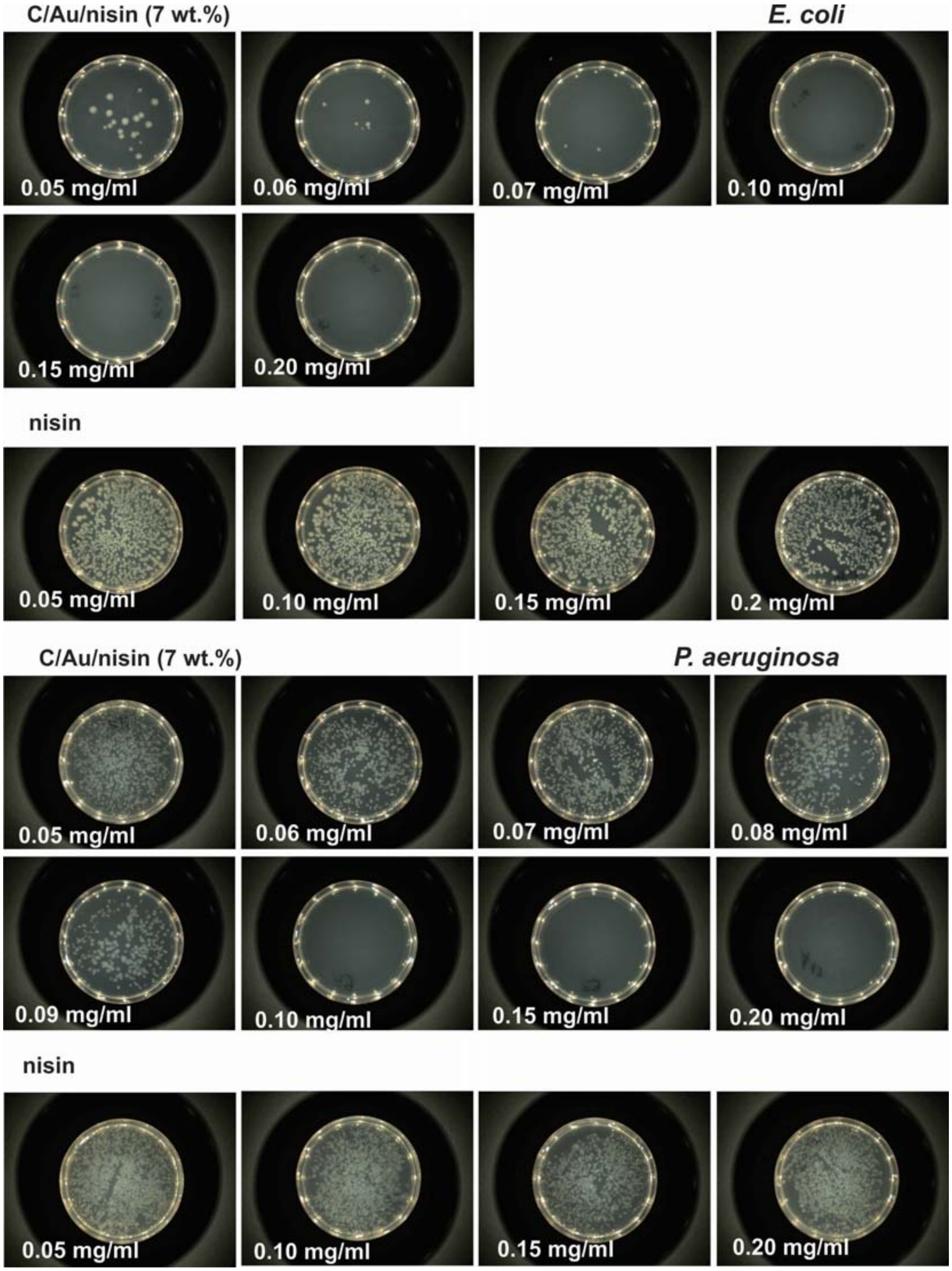

Fig. S4: Determination of the MBC of C/Au/nisin (7 wt.%) against E. coli and P. aeruginosa and comparison of the activity to nisin without functionalization.

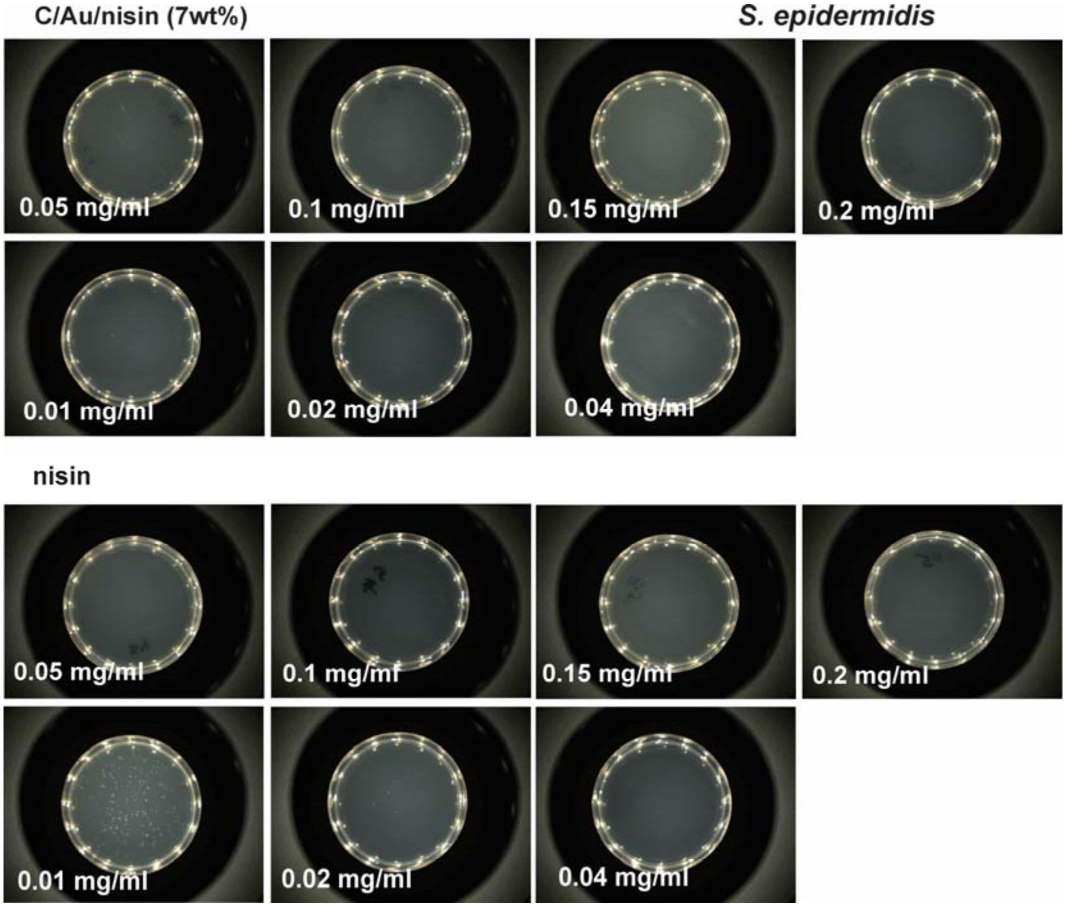

Fig. S5: Determination of the MBC of C/Au/nisin (7 wt.%) against S. epidermidis and comparison of the activity to nisin without functionalization.

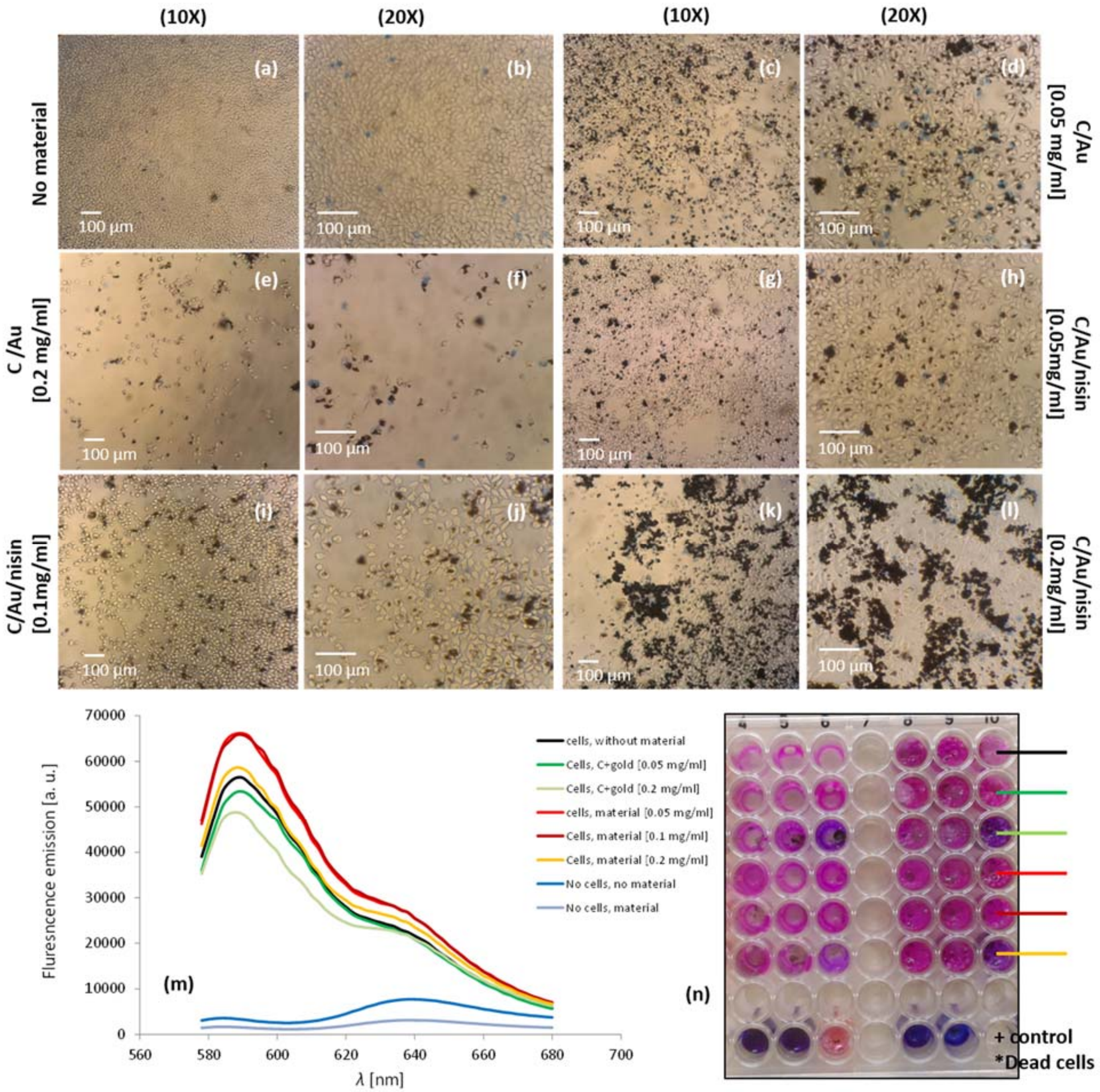

Fig. S6: Tripan blue test- morphological characteristics of tested L929 fibroblasts: reference cells without material (a,b), C/Au at 0.05 mg/ml (c,d) and 0.2 mg/ml (e,f) and C/Au/nisin (containing 7 wt.% of pure nisin) at 0.05 mg/ml (g,h), 0.1 mg/ml (i,j) and 0.2 mg/ml (k,l); Resazurin test- fluorescence spectra for references and cells exposed to materials (m), the same staining for cells with and without materials (n) confirming high viability.

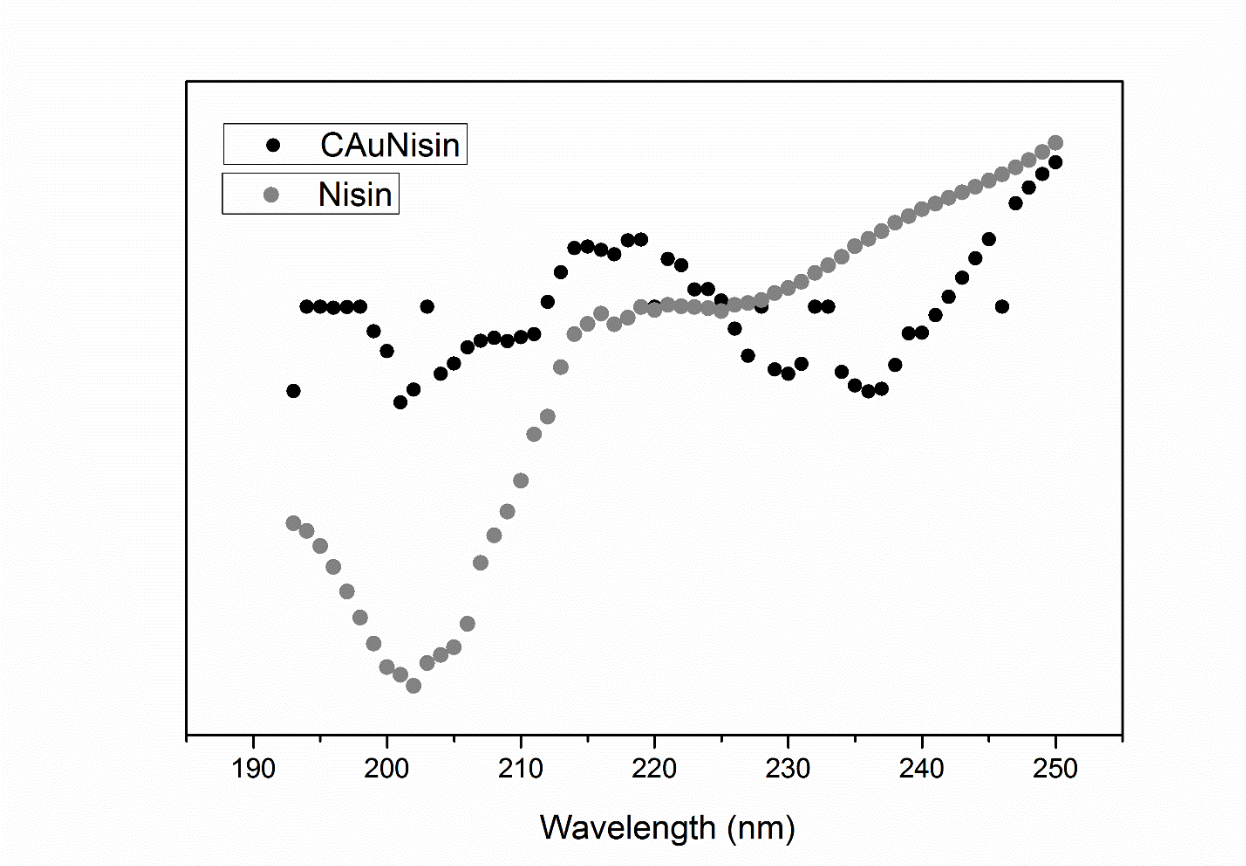

Fig. S7: Circular dichroism spectra of pure nisin and nisin within C/Au composite.
